# Attachment Dimensions and Spatial Navigation in Female College Students: The Role of Comfort With Closeness and Confidence in Others

**DOI:** 10.3389/fpsyg.2019.00235

**Published:** 2019-02-19

**Authors:** Nuno Barbosa Rocha, Andreia Lemos, Carlos Campos, Susana Rocha, Tetsuya Yamamoto, Sérgio Machado, Eric Murillo-Rodriguez

**Affiliations:** ^1^Center for Rehabilitation Research, School of Health, Polytechnic Institute of Porto, Porto, Portugal; ^2^School of Accounting and Administration, Polytechnic Institute of Porto, Porto, Portugal; ^3^Graduate School of Technology, Industrial and Social Sciences, Tokushima University, Tokushima, Japan; ^4^Physical Activity Neuroscience Laboratory, Physical Activity Sciences Postgraduate Program, Salgado de Oliveira University, Niterói, Brazil; ^5^Laboratorio de Neurociencias Moleculares e Integrativas, Escuela de Medicina, División Ciencias de la Salud, Universidad Anáhuac Mayab, Mérida, Mexico

**Keywords:** attachment, spatial learning, spatial navigation, hippocampus, spatial recall

## Abstract

There is preliminary evidence suggesting that hippocampal functioning is associated with attachment style. However, it is unknown if attachment is also associated with hippocampal-related cognitive function such as spatial learning and recall. This study aims to verify if attachment dimensions are associated with spatial learning and recall. Sixty-five female participants were recruited and were evaluated using the Adult Attachment Scale-R and tested on a virtual maze navigation task (VMT) at one moment (exploratory trial + 3 trials) and 24 h later (3 trials). There was a significant Moment × Trial × Close-Depend interaction for the outcome time, *F*(2,126) = 3.807, *p* = 0.025, with *post hoc* analysis indicating that the High Close-Depend group displayed significant improvements between Trial 1 and Trial 3 in the post-test assessment. Conversely, the Low Close-Depend group displayed significant improvements between Trial 1and Trial 3 but on the pre-test assessment. Furthermore, the Low Close-Depend group presented significant better performance in pre-test Trial 3 in comparison to the High Close-Depend group. Thereby, it seems that low comfort with proximity and trust in others is associated with reduced spatial recall, although spatial learning performance was actually superior in these participants. It is possible that reduced exposure to social interaction and meaningful relationships may be reduced in the Low Close-Depend group, leading to modifications in hippocampal function and, ultimately, reduced spatial recall. Oppositely, participants in the High Close-Depend group may not display typical spatial learning in the proposed task as they are more willing to freely explore the presented environment.

## Introduction

John Bowlby defined attachment as a deep and lasting emotional relationship that links one person to another in time and space (Gully, 2014). There are several models that propose different classifications for attachment styles (Hazan and Shaver, 1987; Ainsworth et al., 2015), but the most widely used one has been proposed by Bartholomew and Horowitz (1991) and categorizes attachment into four styles: secure, anxious-preoccupied, dismissive-avoidant and fearful-avoidant. Secure individuals have a positive view of themselves and others, and display a balance between emotional proximity and dependence in relationships. Conversely, anxious-preoccupied individuals have a negative view of themselves and display a need for emotional closeness, as well as an excessive fear of abandonment and reduced proximity avoidance. Dismissive-avoidant individuals feel uncomfortable with intimate relationships and avoid them. They also overvalue the sense of independence and self-sufficiency. Lastly, fearful-avoidant individuals have a negative view of themselves, have difficulty to trust others and feelings of inadequacy or personal vulnerability that distract them from others (Bartholomew and Horowitz, 1991; Canavarro et al., 2006). The previously described categorization is actually developed using a three-factor measure of adult attachment that encompasses the following dimensions (Collins and Read, 1990): Depend, referring to the extent to which a person feels he/she can depend/trust on the other to be available whenever needed; Anxiety, worry regarding being rejected or unloved; Close, representing the extent to which someone feels comfortable with closeness and intimacy. However, there is a long-lasting debated questioning whether attachment is more effectively comprehended using categorical or continuous/dimensional models. Recently it has been become widely accepted that using dimensional models can be a more effective strategy to conceptualize and measure individual differences in attachment (Fraley et al., 2015).

In the last decade there have been several studies exploring the neurobiological underpinnings of attachment. Quirin et al. (2009) reported that insecure attachment styles are associated with reduced gray matter density in the hippocampus which main possibly disturb the regulation stress. Gray matter density in brain structures related to emotion regulation is reportedly reduced in major depression and post-traumatic stress disorder (Lee et al., 2011; Zhang et al., 2011). Also, individuals with insecure attachment styles typically display increased anxiety levels, less effective regulatory strategies and enhanced cortisol production in high stress situations in comparison to subjects with secure attachment (Nolte et al., 2011). Findings regarding cortical levels in subject with dismissive attachment style are somewhat inconsistent (Dewitte et al., 2010; Jaremka et al., 2013). Finally, it is important to highlight that the evidence exploring the biological basis of attachment using a dimensional approach is extremely scarce. Regardless, there is preliminary evidence suggesting that hippocampal functioning is associated with attachment style.

It is also interesting to postulate whether attachment style can incrementally induce neurobiological changes in individuals across their life span. For instance, anxious-preoccupied and fearful-avoidant individuals are more likely to be exposed to situations of chronic stress, which can deregulate HPA axis activity, cortisol overexpression and subsequent morphological changes in neurons, namely hippocampal cells (Kim et al., 2015; Ebner and Singewald, 2017). Furthermore, the hippocampus actually plays an important role in the HPA axis inhibition, which can lead to a positive feedback loop that increases the production of glucocorticoids (Smith and Vale, 2006; Zhu et al., 2014). These pathways are in line with the glucocorticoid vulnerability hypothesis that states that chronic stress and increased glucocorticoid expression assume a critical role in the susceptibility of the hippocampus to suffer aggressions and degenerative processes (Conrad, 2008).

Since the discovery of place cells in animal models (Moser et al., 2015), the hippocampus has been highlighted as a critical structure for spatial learning and recall. Hippocampal lesions have been associated with deficits in maintaining a sense of direction and location while the individual moves in a given environment (Ramos, 2009; Eichenbaum and Cohen, 2014). Thereby, as hippocampal functioning has been associated with attachment, it seems reasonable to assume that spatial navigation may also be related with this construct. Understanding the association between attachment dimensions and spatial navigation may shed light on the neurocognitive deficits experienced by subjects exposed to life situation that are critical for attachment (e.g., social deprived children). Thus, this study aims to verify if attachment dimensions are associated with spatial learning and recall in adult female college students.

## Materials and Methods

### Participants

Sixty-five female undergraduate students from the School of Health – Polytechnic of Porto were recruited. Only female participants were included since there are well-known differences in spatial navigation performance between men and women and the number of male subjects available for participation was scarce. Participants reported no history of psychiatric, sleep, and neurological disorders as well as psychotropic medication intake. Participants were also instructed to abstain from caffeine, alcohol, and/or drug use for 24 h prior to the study. Included subjects did not have regular experience playing 3D or first-person perspective computer gaming.

### Instruments

The Portuguese version of the Adult Attachment Scale – R (Collins and Read, 1990) was used to assess attachment style in three dimensions: Close (comfort with closeness); Depend (trust in others), and Anxiety (related to abandonment). For scoring purposes, the average score for each dimension was calculated. Subsequently, the average score of Close and Depend was computed to create and new dimension named Close-Depend. Therefore, participants were categorized and allocated to several groups based on two dimensions: High vs. Low Anxiety Groups as well as High vs. Low Close-Depend Groups. The Virtual Maze Task (VMT) was used to evaluate spatial navigation (Nguyen et al., 2013). This task consisted of a maze with a 20 × 20 grid units structure constructed using the Unreal Tournament 3 Editor (Epic Games, Cary, NC, United States), in which participants had to find the exit. There was an initial exploratory trial where participants started in the exit and had 5 min to explore the maze. In the following three trials, participants were asked to find the exit door as quickly as possible, starting from three random positions in the maze. Each trial ended when the individual reached the exit or after a maximum period of 10 min. Performance was assessed by three outcome measures: time to complete the task (in seconds), number of moves and backtracking during maze navigation. Number of moves was computed by measuring the number of grid units where the participant moved. Backtracking is the ratio of unique positions (grids where the participant only moved once) by the total number of moves. Reduced backtracking suggests that the participant was more disoriented during task performance.

### Procedures

The study was approved by the Ethics Committee of the School of Health of the Polytechnic Institute of Porto. Written informed consent was obtained for each participant. In the first visit to the laboratory, participants completed a demographic questionnaire, the Adult Attachment Scale – R and the pre-test VMT. The pre-test VMT encompassed a 5-min exploratory trial to get acquainted with the maze, followed by three trials in which participants have to find the maze exit as fast as they can. Each participant visited the laboratory 24 h after the initial assessment in order to complete the post-test VMT, in which they completed again three trials of the maze without the initial exploration phase. Thus, participants completed the VMT task in two distinct moments. While the pre-test VMT aimed to assess spatial learning, the 24 h delayed post-test VMT was used to assess spatial navigation recall.

For data analysis participants were grouped based on the Anxiety and Close-Depend dimensions of the Adult Attachment Scale – R and separate analysis were completed for each dimension. Thus, participants with average Anxiety scores higher than 3 were placed on the High Anxiety Group, whilst subjects with scores lower than three were attributed in the Low Anxiety Group. Similarly, participants with average Close-Depend scores higher than 3 were placed in the High Close-Depend group and participants with scores lower than 3 were designated to the Low Close-Depend group.

Statistical analysis was performed using the Statistical Package for Social Sciences (SPSS) 24.0. Three-way mixed ANOVAs with two within-group factors (Moment and Trial) and one between-group factor (Attachment Groups) were used to analyze effects on spatial navigation outcomes (distance, time and backtracking). For each outcome, two analyzes were carried out based on different Attachment Groups: Anxiety criteria (High Anxiety vs. Low Anxiety:) and Close-Depend criteria (High Close-Depend vs. Low Close-Depend). Before each analysis, the assumptions of normality (Shapiro–Wilk test and asymmetry and kurtosis values), homogeneity of variances (Levene test) and sphericity (Mauchly test) were tested (Colwell, 2006; Marôco, 2014). A *post hoc* analysis was also performed for each test using the Bonferroni test. Independent samples *t*-test and Mann–Whitney test were used to compare Attachment Groups regarding age and Adult Attachment Scale – R subscores. The Mann–Whitney test was used for variables that did not present a normal distribution. For the independent samples *t*-test the correction for heterogeneity of variance was used in variables that did not fulfill the assumption of homogeneity. All statistical tests were completed with a significance level of 0.05.

## Results

Table 1 displays total sample characteristics (age and attachment subscores) as well as characterization for Attachment Groups. The High Anxiety Group was composed by 32 individuals, whilst the Low Anxiety Group had 33 subjects. For the Close-Depend dimension, the High Close-Depend Group included 43 participants and the Low Close-Depend Group had 22 subjects. There were no significant differences in the Close-Depend group comparison for any of the analyzed variables (*p* > 0.05), except for the Close, Depend and Close-Depend scores (which were firstly used to create these groups). However, in the Anxiety groups comparison there was a significant difference in age between the two groups, *U* = 286.000 and *p* = 0.001. Thereby, an ANCOVA *a posteriori* was also performed for each analysis related to Anxiety in order to verify if age had any effect on the reported findings.

**Table 1:** Sample characterization.

	Total sample (*n* = 65)	High anxiety (*n* = 32)	Low anxiety (*n* = 33)	Between group comparison	High Close-Depend (*n* = 43)	Low Close-Depend (*n* = 22)	Between group comparison
	
	*M* (s.d.)	*M* (s.d.)	*M* (s.d.)	*p*	*M* (s.d.)	*M* (s.d.)	*p*
Age	19.98	19.31	20.64	0.001*	20.02	19.91	0.874*
	(1.99)	(1.47)	(2.22)		(2.13)	(1.72)	
Anxiety	2.79	3.60	2.00	0.000	2.67	3.02	0.078**
	(0.92)	(0.40)	(0.48)		(1.04)	(0.57)	
Close	3.48	3.42	3.55	0.322	3.75	2.96	<0.001
	(0.54)	(0.47)	(0.60)		(0.43)	(0.31)	
Depend	3.15	3.02	3.28	0.080	3.44	2.59	<0.001
	(0.62)	(0.67)	(0.54)		(0.50)	(0.40)	
Close-Depend	3.32	3.22	3.42	0.102	3.60	2.78	<0.001**
	(0.49)	(0.47)	(0.51)		(0.36)	(0.13)	


*^∗^Mann–Whitney test; ^∗∗^Student’s *t*-test with correction for heterogeneity of variances*.

The Shapiro–Wilk normality tests indicated that there are multiple variables in which at least one of the groups under analysis did not display a normal distribution (*p* < 0.05). However, simulation studies suggest that ANOVA is robust enough to compare samples with non-normal distributions, with skewness values up to |2.0| and kurtosis up to |9.0| (Ito, 1980; Marôco, 2014). Thus, although several outcomes did not present a normal distribution in one or more of the groups under analysis, the skewness and kurtosis values were clearly lower than those mentioned above, allowing for a valid interpretation of the ANOVA results.

Regarding the Levene tests, there was only heterogeneity of variances in the distance completed on Trial 3 of pre-test VMT, when participants were grouped by the Close-Depend dimension (*p* = 0.007). However, as all the other outcomes under analysis met the assumption of homogeneity and there is evidence that ANOVA is robust to slight homogeneity violations (Ito, 1980), it can be assumed that the analyzes that included this variable are reliable. It should also be noted that the sphericity assumption (Mauchly test) was met for the ANOVA analysis. Following, the ANOVA results for each spatial navigation outcome will be presented.

### Time to Complete the Maze

**FIGURE 1:**
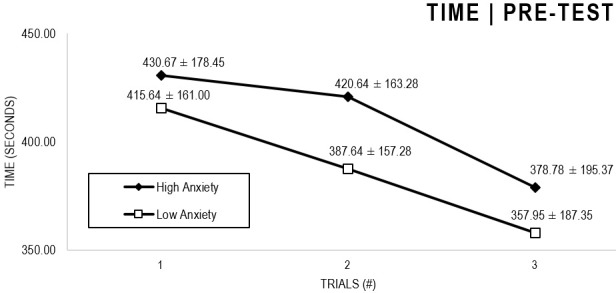
Outcome time: High and Low Anxiety groups comparison (pre-test).

**FIGURE 2:**
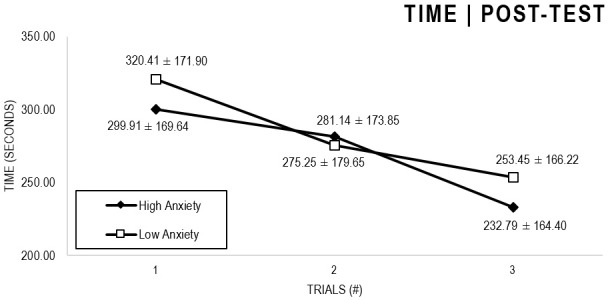
Outcome time: High and Low Anxiety groups comparison (post-test).

In the Anxiety analyses (Figure 1, 2), there were significant main effects for Moment, *F*(1,63) = 64.422, *p* < 0.001, and Trial, *F*(1,63) = 6.848, *p* = 0.002, as participants displayed better performance on post-test VMT in comparison to pre-test, and on Trial 3 in comparison to Trial 1 (*p* = 0.002). There were no significant main effects for Close-Depend Groups, *F*(1,63) = 0.035, *p* = 0.851. Finally, there were no significant interactions for Moment × Trial × Group, *F*(2,126) = 0.024, *p* = 0.976, Moment × Trial, *F*(2,126) = 0.100, *p* = 0.905, Moment × Group, *F*(1,63) = 1.317, *p* = 0.256, and Trial × Group, *F*(2,126) = 0.267, *p* = 0.766. There were no change in the significance of *p*-values for the aforementioned interactions after adding age as covariate.

**FIGURE 3:**
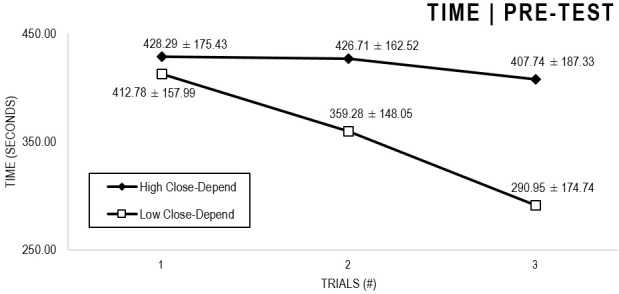
Outcome time: High and Low Close-Depend groups comparison (pre-test).

**FIGURE 4:**
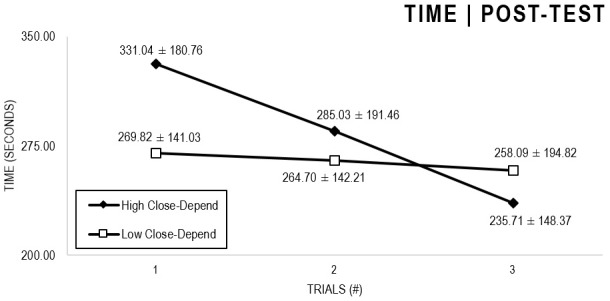
Outcome time: High and Low Close-Depend groups comparison (post-test).

In the Close-Depend analyses (Figure 3, 4), there were significant main effects for Moment, *F*(1,63) = 51.178, *p* < 0.001, and Trial, *F*(1,63) = 6.397, *p* = 0.002, as participants displayed better performance on post-test VMT in comparison to pre-test and on Trial 3 in comparison to Trial 1 (*p* = 0.003). There were no significant main effects for Close-Depend Groups, *F*(1,63) = 1.937, *p* = 0.160. Furthermore, there was a significant Moment × Trial × Group interaction, *F*(2,126) = 3.807, *p* = 0.025, although there were no significant interactions for Moment × Trial, *F*(2,126) = 0.167, *p* = 0.846, Moment × Group, *F*(1,63) = 2.178, *p* = 0.145, and Trial × Group, *F*(2,126) = 0.033, *p* = 0.968. The *post hoc* analysis indicated a significant difference in the High Close-Depend Group (*p* = 0.002) between Trial 1 (*M* = 331.04) and Trial 3 (*M* = 235.71) of the post-test VMT. Conversely, in the Low Close-Depend Group, the significant difference (*p* = 0.023) between Trial 1 (*M* = 412.78) and Trial 3 (*M* = 290.95) occurred in the pre-test VMT. In addition, there was a significant difference (*p* = 0.018) between the Close-Depend Groups in Trial 3 in the pre-test VTM (High Comfort-Trust = 407.74; Low Comfort-Trust = 290.95).

### Number of Moves

**FIGURE 5:**
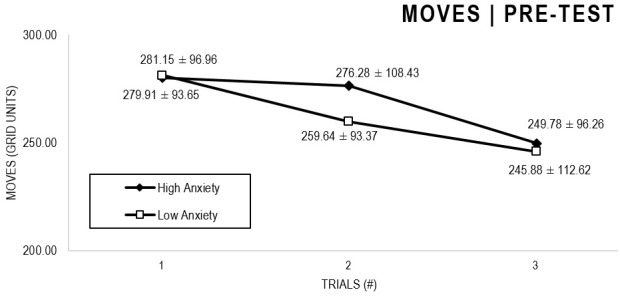
Outcome number of moves: High and Low Anxiety groups comparison (pre-test).

**FIGURE 6:**
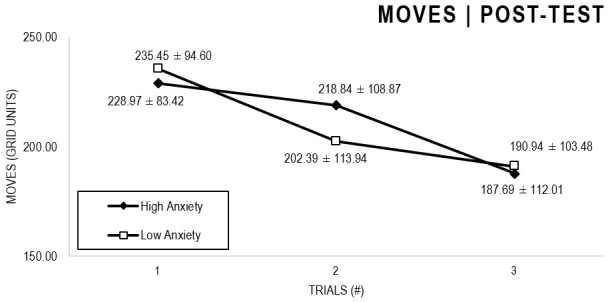
Outcome number of moves: High and Low Anxiety groups comparison (post-test).

In the Anxiety analyses (Figure 5, 6), there were significant main effects for Moment, *F*(1,63) = 27.796, *p* < 0.001, and Trial, *F*(1,63) = 5.485, *p* = 0.005, as participants displayed better performance on post-test VMT in comparison to pre-test, and on Trial 3 in comparison to Trial 1 (*p* = 0.009). There were no significant main effects for Anxiety Groups, *F*(1,63) = 0.090, *p* = 0.765. There were no significant interactions for Moment × Trial × Group, *F*(2,126) = 0.015, *p* = 0.986, Moment × Trial, *F*(2,126) = 0.140, *p* = 0.869, Moment × Group, *F*(1,63) = 0.041, *p* = 0.840, Trial × Group, *F*(2,126) = 0.445, *p* = 0.642. There were no change in the significance of *p*-values for the aforementioned interactions after adding age as covariate.

**FIGURE 7:**
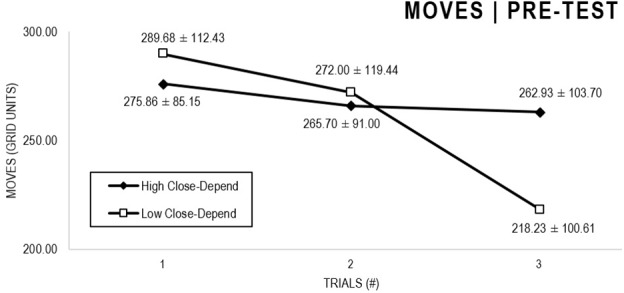
Outcome number of moves: High and Low Close-Depend groups comparison (pre-test).

**FIGURE 8:**
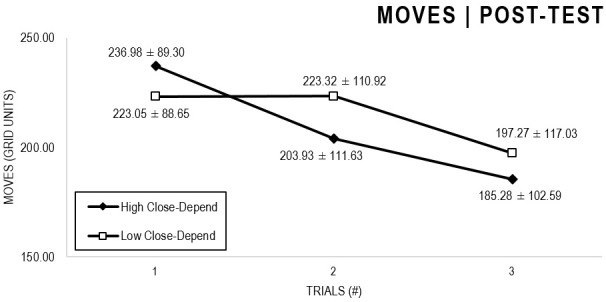
Outcome number of moves: High and Low Close-Depend groups comparison (post-test).

In the Close-Depend analyses (Figure 7, 8), there were significant main effects for Moment, *F*(1,63) = 22.989, *p* < 0.001, and Trial, *F*(1,63) = 5.758, *p* = 0.004, as participants displayed better performance on post-test VMT in comparison to pre-test and on Trial 3 in comparison to Trial 1 (*p* = 0.007). There were no significant main effects for Close-Depend Groups, *F*(1,63) = 0.006, *p* = 0.938. There were no significant interactions for Moment × Trial × Anxiety Group, *F*(2,126) = 1,854, *p* = 0.161, Moment × Trial, *F*(2,126) = 0.037, *p* = 0.964, Moment × Group, *F*(1,63) = 0.410, *p* = 0.524, and Trial × Group, *F*(2,126) = 0.737, *p* = 0.480.

### Backtracking

**FIGURE 9:**
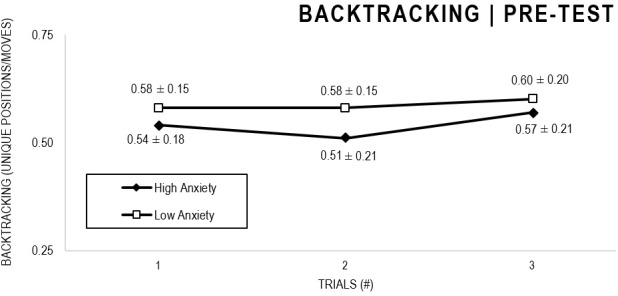
Outcome backtracking: High and Low Anxiety groups comparison (pre-test).

**FIGURE 10:**
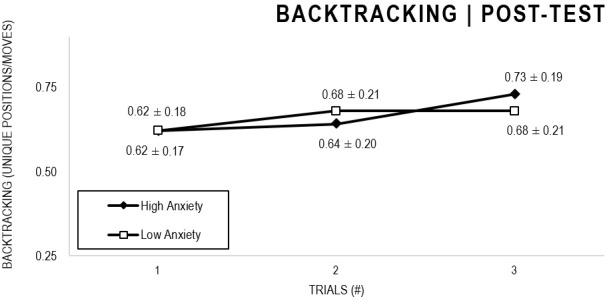
Outcome backtracking: High and Low Anxiety groups comparison (post-test).

In the Anxiety analyses (Figure 9, 10), there were significant main effects for Moment, *F*(1,63) = 30.546, *p* < 0.001, and Trial, *F*(1,63) = 3.764, *p* = 0.026, as participants displayed better performance on post-test VMT in comparison to pre-test, and on Trial 3 in comparison to Trial 1 (*p* = 0.028). There were no significant main effects for Anxiety Groups, *F*(1,63) = 0.615, *p* = 0.436. There were no significant interactions for Moment × Test × Anxiety Group, *F*(2,126) = 0.198, *p* = 0.821, Moment × Trial, *F*(2,126) = 1.007, *p* = 0.368, Moment × Group, *F*(1,63) = 1.719, *p* = 0.195, Trial × Group, *F*(2,126) = 0.843, *p* = 0.433. There were no change in the significance of *p*-values for the aforementioned interactions after adding age as covariate.

**FIGURE 11:**
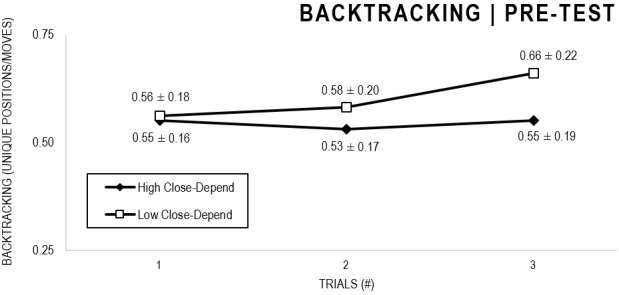
Outcome backtracking: High and Low Close-Depend groups comparison (pre-test).

**FIGURE 12:**
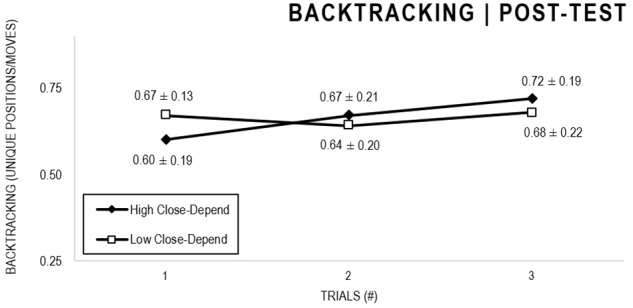
Outcome backtracking: High and Low Close-Depend groups comparison (post-test).

In the Close-Depend analyses, there were significant main effects for Moment, *F*(1,63) = 22.602, *p* < 0.001, and Trial, *F*(1,63) = 3.416, *p* = 0.036, as participants displayed better performance on post-test VMT in comparison to pre-test and on Trial 3 in comparison to Trial 1 (*p* = 0.044). There were no significant main effects for Close-Depend Groups, *F*(1,63) = 0.973, *p* = 0.328. There was also a significant interaction between Moment × Trial × Group, *F*(2,126) = 3,206, *p* = 0.044, although there were no significant interactions for Moment × Trial, *F*(2,126) = 0.186, *p* = 0.830, Moment × Group, *F*(1,63) = 2.171, *p* = 0.146, Trial × Group, *F*(2,126) = 0.167, *p* = 0.846. The *post hoc* analysis indicated a significant difference in the High Close-Depend Group (*p* = 0.003) between Trial 1 (*M* = 0.601) and Trial 3 (*M* = 0.719) of the post-test VMT. In the Low Close-Depend Group there were no significant differences between trials in both pre-test and post-test VMT (*p* > 0.05). However, there was a significant difference (*p* = 0.038) between the Close-Depend Groups in Trial 3 in the pre-test VTM (High Close-Depend = 0.549; Low Close-Depend = 0.658; Figure 11, 12).

## Discussion

This study aimed to explore the association between adult attachment dimensions and spatial navigation (learning and recall) in adult female college students. Our results suggest that spatial learning and recall is not associated to the Anxiety attachment dimension, but rather to the Close-Depend domain. More specifically, participants with low levels of Close-Depend scores, usually associated with frightened and dismissive attachment, displayed reduced spatial recall in comparison to the high Close-Depend group, typically associated with secure attachment styles. It is known that individuals with higher stress levels as seen in insecure attachment styles present a higher secretion of cortisol compared to secure individuals. Long-term over-production of cortisol may lead structural and physiological changes in the hippocampus (Dewitte et al., 2010; Jaremka et al., 2013; Kim et al., 2015) that can ultimately impair participant’s performance on spatial navigation tasks. Efficient spatial navigation requires the integrity of the two hippocampal formations as they display distinct but complementary roles (Maguire et al., 2000; Fanselow and Dong, 2010).

Another key factor that can explain reduced hippocampal-dependent spatial recall in subjects with low Close-Depend scores is reduced exposure to social interaction throughout their life. As social beings, humans benefit from the contact with other people. Social interaction allows the brain to be stimulated and to maintain proper functional properties. Thus, when an individual is rarely engaged in social contacts and relationships, typical brain functioning may be compromised (Council, 2015; Takeuchi et al., 2015). Several suggest that the hippocampus is one of the brain structures highly sensitive to environmental and psychosocial factors (Tottenham and Sheridan, 2009; McEwen et al., 2012). In fact, a study by Luby et al. (2012) with children revealed that maternal support positively influenced the development of the hippocampus. Additionally, Akillioglu et al. (2015) found that rodents deprived from contact with the progenitor showed changes in the hippocampus as well as impaired spatial memory. In addition, a study accomplished by Karimian and Attari (2015) reported that in a sample of twenty male rats, neurogenesis in the dentate gyrus of the hippocampus was significantly lower in isolated rats when compared to the group of social rats. Further studies with animal models also suggest that rodents exposed to long-term social isolation display decreased BDNF expression in the hippocampus, although the mechanisms underlying this results are not known (Eckert and Abraham, 2012; Murínová et al., 2017). BDNF is a neurotrophic growth factor that plays a crucial role in cell proliferation, migration and phenotypic differentiation, maintenance of neuronal functions, structural integrity of neurons and neurogenesis.

The results reported here also suggest that subjects with lower Close-Depend actually presented greater spatial learning abilities as they significantly improved between trials in the pre-test assessment in comparison the high Close-Depend group. These opposite findings on spatial learning may be explained by a two-fold hypothesis. First, it is known that individuals with insecure attachment are more prone to social stress and less effective to regulate their emotions effectively (Ooi et al., 2006; Movahed Abtahi and Kerns, 2017). This vulnerability to stress has been related to HPA hyperactivation as well as enhanced production of cortisol (Dewitte et al., 2010; Jaremka et al., 2013). Moreover, there is evidence suggesting that acute exposure to stress does not impair spatial learning and may even lead to improved performance on these tasks (Duncko et al., 2007).

Thereby, it can be hypothesized that insecure individuals display a biological hyperactivity to stress that actually leads to enhanced spatial learning. Klopp et al. (2012) found that acute social stress does not cause changes in spatial learning performance in college students. Conversely, as the neuronal mechanisms underlying learning and memory after stress are distinct and not affected in the same way, it may possible that social stress actually selectively impairs the recall of spatial memories and not the immediate learning performance.

The second explanatory mechanism for the reported results regarding spatial learning suggest that individuals with high scores on the Close-Depend dimension may have not displayed improvements in pre-test trials as they were more willing to explore the presented maze, without the urge to complete the task as fast as possible. There is significant evidence suggesting that in stress situations, secure individuals are more likely to explore unusual environments. In a study conducted by Main (1983), children with secure attachment (high Close-Depend) showed longer attention span and more intense playful exploration than children with insecure attachment (low Close-Depend). More recently, Ainsworth et al. (2015) found that a child with a higher level of trust in maternal support feels the presence of the mother as comforting, making her feel safe enough to explore the environment. Recent evidence from a report with 90 children, aged 8 to 12 years, suggests that children with secure attachment are better able to freely explore a slightly threatening environment when they mother is present (Dujardin et al., 2015). Conversely, Stupica et al. (2011) showed that insecure attachment traits may undermine the exploratory efforts of children who are more reactive and easily irritated by environmental changes. Together, the previously described studies may help to understand why the High Close-Depend group did not present a significant improvement during the first three trials at pre-test assessment, since subjects prefer a more exploratory behavior than the Low Close-Depend group.

This study presents some limitations that do not allow to generalize these findings. First, the sample size is reduced. Second, it only includes female subjects and spatial navigation performance is typically different between genders which may play a role on the reported findings. Future studies should explore spatial learning and recall in male subjects in order to understand if the reported interactions are similar or somewhat different. Third, this study only included young adults (college students) and does not account for the role of age in the hypothesis. If subjects with decreased comfort with closeness and trust with others are less exposed to social interactions throughout their life it is possible that spatial recall performance is actually modified across time. Finally, as this study is cross-sectional, it does not allow to establish a causal relationship between attachment and spatial learning and recall. Longitudinal studies with children or teenagers could be interesting to understand whether attachment dimensions gradually change spatial navigation performance over the years. Studies with children typically deprived from social contact could also provide further insights regarding this topic.

## Conclusion

This study suggests that there are no significant differences in spatial learning and recall between subjects with high and low anxiety, suggesting that this dimension of attachment does not play a role in spatial navigation performance. However, it was observed that the merged dimension Close-Depend was associated with both spatial learning and recall in young female adults. The high Close-Depend group displayed enhanced spatial recall in comparison to participants with low Close-Depend. Conversely, participants with low Close-Depend presented superior spatial learning on the pre-test assessment. These findings suggest the need for further studies exploring the association between attachment dimensions and hippocampal-dependent learning tasks. Exploring the neurocognitive and neurobiological mechanisms of attachment may help to develop psychological interventions that address the needs of subjects exposed to experiences associated with dysfunctional attachment styles.

## Author Contributions

NR was responsible for the conceptualization of the research, formulated the experimental design, and oversaw the data collection. AL and CC conducted the data collection. NR, AL, CC, and SR formulated and conducted the data analysis and wrote the first draft of the manuscript. SM, TY, and EM-R revised the experimental design, provided the methodological support and contributed to writing the manuscript. All authors revised the whole paper and approved the final version of the manuscript.

## Conflict of Interest Statement

The authors declare that the research was conducted in the absence of any commercial or financial relationships that could be construed as a potential conflict of interest. The reviewer LN-J declared a past collaboration with the authors NR, SM, and EM-R to the handling Editor.
